# Entomological investigation of Japanese encephalitis outbreak in Malkangiri district of Odisha state, India

**DOI:** 10.1590/0074-02760170499

**Published:** 2018-05-14

**Authors:** Sudhansu Sekhar Sahu, Smrutidhara Dash, Thankachy Sonia, Subramaniam Muthukumaravel, Thirumal Sankari, Kasinathan Gunasekaran, Purushothaman Jambulingam

**Affiliations:** Vector Control Research Centre, Indian Council of Medical Research, Medical Complex, Indira Nagar, Puducherry, India

**Keywords:** Japanese encephalitis, outbreak, entomological investigation, Malkangiri, Odisha, India

## Abstract

**BACKGROUND:**

A severe outbreak of Japanese encephalitis (JE) and acute encephalitis syndrome (AES) with high case fatality was reported from Malkangiri district of Odisha state, India during September to November 2016 affecting 336 children with 103 deaths.

**OBJECTIVES:**

The purpose of this study was to investigate the outbreak in the light of entomological determinants.

**METHODS:**

Entomological investigation was carried out in 48 villages from four mostly affected Community Health Centres (CHCs) of Malkangiri district. Dusk collections of resting adults was done in villages from indoor and outdoor sites to record the density of mosquito species, including the known JE vectors, feeding behaviour, parity, dusk index and infection status with JE virus (JEV).

**FINDINGS:**

The per man hour density and dusk index of JE vector species varied from 2.5 to 24.0 and 0.81 to 7.62, respectively in study villages. A total of 1136 mosquitoes belonging to six vector species were subjected to PCR and one pool of *Culex vishnui* was found to be positive for JEV.

**CONCLUSION:**

The JE transmission in Malkangiri district was confirmed. Thorough screening of human blood samples of JE/AES suspected cases and JE vector mosquitoes for the presence of JEV during rainy season every year is recommended.

Japanese encephalitis (JE) is a common mosquito borne flaviviral encephalitis and one of the leading forms of viral encephalitis covering a population of over three billion (Ghosh and Basu 2009). It is one of the most important forms of outbreak and sporadic encephalitis in the tropical regions of Asia, including Japan, China, Taiwan, Korea, Philippines, all of South eastern Asia, and India (Solomon 1997). Japanese encephalitis virus (JEV) is maintained in a zoonotic cycle, which can be both enzootic and epizootic. This cycle involves pigs as the major reservoir/amplifying host, water birds as carriers and mosquitoes as vectors. The *Culex vishnui* subgroup of mosquitoes consisting of *Cx. tritaeniorhynchus*, *Cx. vishnui* and *Cx. pseudovishnui* have been implicated as major vectors of JE in different parts of India (NVBDCP 2018). Outbreaks of JE are reported from many parts of India, and it is considered a major paediatric problem. The first recognition of JE based on serological surveys was in 1955, in Tamil Nadu, India (Namachivayam and Umayal 1982). A total of approximately 65 cases were reported between 1955 and 1966 in Southern India (Carey et al. 1968). Subsequent surveys carried out by the National Institute of Virology of Pune indicated that approximately half of the population in Southern India has neutralising antibodies to the virus. Since 1955, many major outbreaks in different parts of the country have been reported. From the state of Odisha in eastern India only one outbreak of JE was reported from Rourkela city of Sundergarh district in 1989 (Vajpayee et al. 1991). Sporadic JE cases have been diagnosed from hospitalised children between 1992 and 1995 (Devi et al. 1996, Dash et al. 2001). Since then, there is no record of JE infection in the state. Malkangiri district of Odisha state reported 9 deaths among children in 2009 due to acute encephalitis syndrome (AES) (Nayak et al. 2016a). During September-November 2012, children with AES followed by 38 deaths were reported from Malkangiri district of Odisha by the state Health Department. In 2014, eight deaths of children were reported due to AES in seven villages of Korkunda and Kalimela Community Health Centres (CHCs) of the district.

Malkangiri district has a long history of high *falciparum* malaria incidence (Anuse et al. 2015, Gunasekaran et al. 2016). From 2010-2016, the annual parasite incidence (API, which is the number of clinical cases per 1000 people per year) in the district ranged from 18.5 to 60.3 (Source: data from the Office of the Chief District Medical Officer, Koraput and Malkangiri). A severe outbreak of viral encephalitis was reported from 9th September to 2nd December 2016 in the Malkangiri district. It was the most severe outbreak occurred in Odisha ever since in the past; 336 children were affected with JE/AES from 187 villages in seven CHCs. The outbreak first started with four cases of encephalitis admitted at District Head Quarters Hospital (DHH), Malkangiri on 9th September 2016. Among the four admitted, two children died on next day. A total of 579 children were admitted in the general fever ward of the DHH Malkangiri with the complaint of fever. Among them, 243 children were cured and discharged next day of their admission. Blood samples of the remaining 336 children were tested and among them 175 were found IgM positive for JE virus by ELISA. Out of the 103 deaths during the outbreak, 37 deaths were due to JE and remaining 66 were due to AES. Among the 336 children suffered from either JE or AES, 233 children were cured and discharged from the hospital. For the first time, such a large number of deaths occurred due to JE/AES in Odisha state.

In view of the severe outbreaks with life loss and on the request of the Health department, Govt. of Odisha, an entomological investigation was carried out in the affected villages during the outbreak period to monitor the abundance of JE vectors, their infection status, risk factors for transmission of JE and to suggest appropriate vector control intervention measures for implementation by the state/district Health Department.

## MATERIALS AND METHODS

Children between the age group four months to 13 years were affected in 187 villages in Malkangiri district. The common clinical presentations were fever, abdominal pain, recurrent vomiting, lethargy, convulsion and loss of consciousness. The main presenting features were sudden onset of convulsions followed by rapid progression to unconsciousness. Most of the affected children showed hypoglycaemia. No symptomatic adult case was admitted during the outbreak period. Blood samples of the 336 children were tested by the IgM antibody capture ELISA (MAC ELISA) by DHH, Malkangiri. This technique was used for the detection of JE virus- specific IgM antibodies using kits acquired from Indian Council of Medical Research-National Institute of Virology (ICMR-NIV), Pune, India. The kits were supplied by National Vector Borne Disease Control Programme (NVBDCP), India. For the first time, such a large number of deaths (n = 103) occurred due to JE/AES in Odisha state.

The seven affected CHCs in the district have 108 Gram Panchayats (GP) and 1,045 revenue villages nearly 25% are inhabited by settlers and the remaining by tribes. The district has a population of 641,385 (2014 census conducted by health department) living in 109,483 households; population density was 106 inhabitants per km^2^. The distance between CHC and different villages ranged from 1 to 35 km. Three seasons, summer (March-June), rainy (July-October) and cold (November-February), are generally prevailing in the district. Malkangiri, lying just south of the Tropic of Cancer, has a tropical climate. During the last five years, the district is warm almost throughout the year with a maximum temperature of 42 to 47°C in summer months. In winter, the temperature is lower, ranging from 13 to 16°C. The relative humidity (RH) varies between 60 and 97% and generally higher during monsoon and post-monsoon months. The district receives rainfall mostly from southwest monsoon during July to October with an average rainfall of 1349.2 mm. The actual rainfall recorded in the district from 2010 to 2015 was 1852.9 mm, 1103.9 mm, 1715.2 mm, 1783.3 mm, 1509.6 mm, and 1771.02, respectively. The month of July is the wettest and the major rivers may get flooded. The district also receives a small rainfall from the retreating monsoon in the month of November.

Entomological investigation was carried out by Indian Council of Medical Research-Vector Control Research Centre (ICMR-VCRC), Puducherry during September 2016 to November 2016, covering 48 villages selected randomly from four CHCs, viz., Korkunda, Mathili, Kalimela and Podia of the Malkangiri district (Fig. 1). The Korkunda CHC has a population of 143,867 living in 29,667 households, Kalimela CHC with 127,819 population in 25,510 households, Mathili CHC with 103,046 population in 21,529 houses and Podia CHC with a population of 55,427 in 11,337 households. Average rainfall in the area in 2016 was 1471.75 mm and the temperature ranged between 16.0 and 42.6°C.

*Mosquito collections* - For containment of the outbreak, fogging (with 5% malathion and 5% cyphenothrin) was initiated in some of the affected villages. Therefore, the mosquito collections were done in the affected villages where fogging was not done and also in villages where one round of fogging was completed. Density of mosquito species, including the known JE vectors and their blood meal source, parity, dusk index and infection rate for JE virus were recorded.

Dusk collections of resting adults indoors (six human dwellings and three cattle sheds) spending five minutes in each human dwelling and 10 min in each cattle shed and outdoors (from sites such as bushes, plantations, standing crops, root-intrices) spending 1 h in each village were done using oral aspirators (hand catches); thereby spending a total of two man hours in each village. All the collected mosquitoes were identified to species level (Barraud 1934). Per man hour density (PMHD) of known JE vector mosquitoes, i.e., number collected per man hour was calculated and recorded for each species. The known JE vector mosquitoes were dissected out and their ovarioles were examined for dilatations to determine the parity. The proportion of parous was determined as the number of parous mosquitoes out of total dissected. The dusk index (DI) was estimated for measuring the density of recognised vectors of JEV, which incorporates proportion of parous female mosquitoes with abundance. This index was calculated by multiplying the PMHD and the proportion of parous females mosquitoes (Mani et al. 1991).

*Vector infection with JE virus* - The unfed and fully gravid female mosquitoes, after dissection for parity, was pooled and kept in sterile 1.5 mL eppendorf tube. A pool containing five mosquitoes (from same species with same gonotrophic condition or from same habitat or same type of collection or same village) was kept in each tube and stored in liquid nitrogen. The mosquito samples in liquid nitrogen were transported to ICMR-VCRC laboratory and stored at −80°C for JE virus detection.

Ninety four pools containing 1136 mosquitoes were subjected to real-time polymerase chain reaction (RT-PCR) for JE virus detection. A pool containing five mosquitoes was homogenised and RNA was extracted using TRI re-agents method (Molecular Research Center, Inc. USA). The whole mosquito tissue was used for RNA extraction by homogenisation. The extracted RNA from five pools (Each PCR pool contains RNA from 25 mosquitoes) was amplified using primers specific to capsid pre membrane region (Liang 2007) of JE virus genome using transcriptor one step RT-PCR kit (Roche Applied Science, Penzberg, Germany). RNA from 25 mosquitoes in a pool for detection of JE virus was standardised previously in ICMRVCRC laboratory. RT-PCR was performed along with negative and positive controls. After the amplification, 675 bp PCR amplicons were separated on 1.5% agarose gel and results was documented using gel documentation system (Gelstan Medicae, India). Based on the number of positive pools for JE virus, infection rate was calculated.[Fig f1]

**Figure f1:**
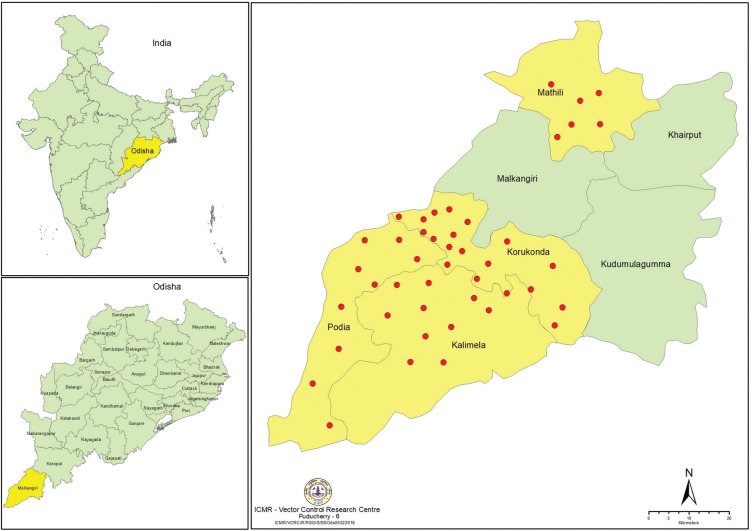
Map showing study villages in Japanese encephalitis (JE) affected community health centres (CHCs) of Malkangiri district.

*Blood meal analysis* - The mid gut of freshly fed JE vector mosquitoes obtained from resting collections was drawn and placed on a circle marked on Whatman No.1 filter paper and squashed to make a round smear, and labelled. Each filter paper with mosquito blood meal smears was kept separately in a self-sealing cover and stored at +4°C until analysis was done for blood meal source using agar gel diffusion method (Crans 1969). The reagents were from MP Biomedicals, (Solon, OH). Before testing the blood meal, the reagents were tested with positive controls to confirm the specificity of the reagents. The mosquito blood-meal was extracted in 0.5 mL saline. A portion of this unknown antigen was placed in each of the two centre wells on a gel plate. Each mosquito blood meal was simultaneously tested for human and bovine hosts by placing the antisera in each of the two surrounding wells. Gel plates were covered with wet chamber petridish and allowed to stand overnight at room temperature. Diffusion of the antigen and antisera took place through the agar so that the blood-meal extract made contact with each of the antisera mid-way between wells. Homologous reactions left a white precipitate band in the agar gel. Tests were read and recorded the following day. The proportion that fed on human and bovine was used to calculate human and bovine blood index (HBI and BBI) for each tested species.

*Statistical analysis* - Data were entered into Microsoft Excel spreadsheet and statistical analyses were carried out using SPSS version 16.0. Pearson correlation test was done to find out if there was a linear association between the mosquito diversity and JE incidence and between the PMHD of major JE vectors and JE incidence. For all statistical tests, the level of significance was taken as p < 0.05.

## RESULTS

*Enzyme-linked immunosorbent assay (ELISA) test with human blood* - Blood samples of the 336 children admitted in the DHH, Malkangiri were tested for the detection of JE virus-specific IgM antibodies and among them 175 were found IgM positive by ELISA. The test was performed by the state Health Department. The JE prevalence among the children admitted was found to be 52.1%. Out of the 103 deaths during the outbreak, 37 deaths were due to JE and remaining 66 were due to AES. While the total (JE/AES) case fatality rate in the current outbreak was 30.7%, the case fatality rate due to JE was 21.1%. Among the 336 children suffered from either JE or AES, 233 children were cured and discharged from the hospital.

*Entomological evaluations* - A total of 14 collections were made spending 28 man-hours in 12 non-fogging villages. In total, 2214 mosquitoes belonging to six known JE vector species were collected. Among them, *Cx. vishnui* (30.4%) was predominant followed by *Cx. whitmorei* (24.9%), *Cx. tritaeniorhynchus* (17.7%), *Cx. bitaeniorhynchus* (13.6%), *Cx. gelidus* (7.9%) and *Cx. fuscocephalus* (5.6%). The PMHD of the JE vector species varied from 4.4 to 24.0. The dusk index ranged from 1.39 *(Cx. gelidus)* to 7.62 (*Cx. vishnui)* among the vectors (Table I).

**TABLE I:** The per man hour density (PMHD), proportion parous (PP) and dusk index (DI) of different Japanese encephalitis (JE) vectors in study villages

		Non fogging				Fogging			
Sl. no	Species	Total collected	PMHD	PP	DI	Total collected	PMHD	PP	DI
1	*Culex bitaeniorhynchus*	301	10.8	0.36	3.85	506	6.49	0.16	1.02
2	*Cx. fuscocephalus*	123	4.4	0.36	1.60	329	4.22	0.26	1.10
3	*Cx. gelidus*	175	6.3	0.22	1.39	193	2.47	0.33	0.81
4	*Cx. tritaeniorhynchus*	392	14 .0	0.38	5.28	648	8.31	0.29	2.40
5	*Cx. vishnui*	672	24.0	0.32	7.62	1 31 2	16.82	0.27	4.53
6	*Cx. whitmorei*	551	19.7	0.30	5.82	1576	20.21	0.31	6.32

A total of 39 collections were made spending 78 man-hours in 36 villages where one round of fogging was completed and 4,564 culicine mosquitoes belonging to six species were collected. Out of the six vector species collected, the proportion of *Cx. whitmorei*, *Cx. vishnui*, *Cx. tritaeniorhynchus*, *Cx. bitaeniorhynchus*, *Cx. fuscocephalus* and *Cx. gelidus* was 34.5%, 28.7%, 14.2%, 11.1%, 7.2% and 4.2%, respectively. The PMHD and dusk index of the six species varied from 2.5 (*Cx. gelidus*) to 20.2 (*Cx. whitmorei*) and 0.81 (*Cx. gelidus*) to 6.32 (*Cx. whitmorei*), respectively in the villages with fogging. The PMHD and dusk index of *Cx. vishnui* was 16.82 and 4.53, respectively (Table I). There was no correlation between the number of vector species and JE incidences (r = −0.013, p = 0.926). Further, correlation analysis indicated that the densities of *Cx. vishnui* (r = −0.048, p = 0.735) and *Cx. tritaeniorhynchus* (r = −0.102, p = 0.465) showed no correlation with JE incidences.

*JE virus detection* - RT-PCR assay was performed in 94 pools containing 1136 mosquitoes belonging to the six vector species collected during the study period and of which, one pool of *Cx. vishnui* was found to be positive for JE virus and the minimum infection rate was found to be 0.88%.

*Blood meal analysis* - A total of 259 samples were analysed for the blood meal source and among them, two samples (HBI-0.008) were found to contain human blood and the remaining were bovine (BBI-0.992). The species found positive for human blood were *Cx. vishnui* (HBI-0.014) and *Cx. whitmorei* (HBI- 0.021) (Table II).

**TABLE II:** Human blood index (HBI) of Japanese encephalitis (JE) vector mosquitoes collected from JE/acute encephalitis syndrome (AES) outbreak villages

Sl. no	Species	Total tested	Number positive to human	HBI
1	*Culex bitaeniorhynchus*	58	0	0
2	*Cx. fuscocephalus*	11	0	0
3	*Cx. gelidus*	15	0	0
4	*Cx. tritaeniorhynchus*	57	0	0
5	*Cx. vishnui*	70	1	0.014
6	*Cx. whitmorei*	48	1	0.021
	Total	259	2	0.008

## DISCUSSION

JE is a viral disease spread by mosquitoes to humans, from infected animals usually pigs and wading birds. Areas with rice fields, where mosquitoes thrive and where there is a lot of pig farming, are especially risky (Bhowmik et al. 2012). JE has been known in India since 1952 (Work and Shah 1956). A total of 63 deaths occurred in Malkangiri from four outbreaks (JE/AES) happened during 2009 to 2014 (Nayak et al. 2016a). All the victims were children. But the recent outbreak was reported to be the most severe one occurred in the state till the end of 2016. The initial stages of the current outbreak evidence pointed to JE because all the cases occurred during monsoon. JE is a disease principally of agricultural areas, particularly in rice cultivation areas, where vector mosquitoes proliferate in close association with pigs, wading birds and ducks. The current study was conducted in rainy and post rainy seasons. Paddy fields are the favourable breeding places during rainy seasons. *Cx. vishnui* subgroup of mosquitoes breed in water with luxuriant vegetation, mainly in paddy fields and their abundance may be related to their breeding in rice fields (Medhi et al. 2017). In the current study, all the JE/AES affected villages were surrounded by paddy fields thereby, providing favourable breeding places for the vector mosquitoes. However, persistence of a high number of cases with similar symptoms for a prolonged period might be due to the existence of another causative agent. Majority of cases showed symptoms of vomiting, dizziness and loss of appetite when they were admitted to the hospital. This led the doctors to suspect that they were suffering from JE/AES, which is characterised by inflammation of brain leading to death in some cases. The present outbreak of JE/AES persisted for three months. The human (175 JEV positive out of 336 samples tested) and pig (18 JEV positive out of 239 samples tested) (Data source: Chief District Veterinary Office, Malkangiri) serological investigation confirmed the occurrence of JEV infection in the Malkangiri district. The current outbreak of viral encephalitis extended to Sukuma district (neighbouring district of Malkangiri) of Chhattisgarh state where five AES cases were admitted in DHH of Malkangiri and three of them died. Another neighbouring district, i.e., Koraput of Odisha, where 62 AES cases were reported during the same period and among them, 29 were found positive for JE virus on testing their serum samples. Among the 62 cases admitted in Koraput DHH, four died due to JE and 10 due to AES.

Entomological investigations revealed the presence of six vector species of JE in the study villages, including the two major vector species viz., *Cx. vishnui* and *Cx. tritaeniorhynchus*, which constituted 44.6% of the total JE vector mosquitoes. The PMHD and the DI of these two vector species were relatively higher in the affected villages where no fogging was done. Since, the same villages could not be monitored for change in density before and after one round of fogging, the differences in mosquito occurrences might not be related to fogging. The detection of JEV in *Cx. vishnui* has important implications for public health and for JE control agenda. This is the first demonstration of detection of JEV from outbreak affected villages of Malkangiri district in Odisha state.

The limitation of the study was that, the present study being an entomological outbreak investigation of JE/AES, the selection of study villages for entomological collections before and after fogging, man hour spent etc. could not be planned beforehand. Since, this was an outbreak condition, fogging was done profusely in affected villages. Hence, same villages could not be monitored for change in density before and after one round of fogging.

During earlier outbreaks occurred in Malkangiri district, the association of JE was confirmed as JE virus IgM from serological samples and JE virus RNA from cerebrospinal fluid (CSF) samples was detected (Dwibedi et al. 2015, Nayak et al. 2016a) and was lacking the supporting entomological confirmations from viral isolation of JE vectors. In the current outbreak, the presence of JEV was confirmed in vector mosquitoes by PCR assay. Further, *Cx. vishnui* was found to have fed on human blood and virus detection was confirmed from the same species. Hence, *Cx. vishnui* could be involved in JE transmission in the current outbreak affected area. Earlier different studies showed that maximum detection of JEV was obtained from the *Culex vishnui* subgroup (Chakravarty et al. 1975, Banerjee et al. 1978, Mourya et al. 1989, Dhanda et al. 1997, Gajanana et al. 1997, Mariappan et al. 2014) and this subgroup mosquitoes were also shown to be capable of transmitting the JE virus in the laboratory (Soman et al. 1977). The current observations on the presence of JEV in *Cx. vishnui* is also in line with the earlier findings observed in Mayurbhanj district of Odisha state (Nayak et al. 2016b). The evidence gathered such as availability of paddy fields, abundance of JE vectors, their dusk index, HBI and infection status in the current investigation pointed towards JE virus aetiology.

Based on the findings of this investigation, appropriate vector control measures were recommended to local general/health administration and they mounted a well-funded campaign to control the outbreak. Intense vector control interventions such as fogging indoors and outdoors with 5% malathion and 5% cyphenothrin, larvicidal (*Bti)* spray in temporary breeding habitats found inside the villages and LLINs/non-impregnated nets were distributed in the affected villages. Five rounds of fogging were carried out in the affected villages up to the mid December 2016. A total of 6200 odomos mosquito repellent creams were distributed by the female health workers to the children of the both affected and nearby affected villages to apply on their body during evening time for preventing from mosquito bites. As JE vector mosquitoes are transmitting the disease from infected pigs to humans, measures were taken by the government and community to keep the pigs in pig pens constructed in the isolated places 2.5 km away from the villages. In addition, the people in affected villages sold nearly 15,000 pigs to neighbouring districts as a means of eliminating the source of the virus. Bush cutting in the affected villages was carried out intensively. All the low lying area where water accumulation occurred was filled with sand and soil. JE vaccines have been administered within a week after control of outbreak (second week of December 2016) to all the children (221,155) below 15 years for preventing the outbreak in future. An active programme of health education and social mobilisation has been mounted in affected areas.

The geographic features of this district support the spread of JEV. Paddy fields of the affected villages are located within 0.3 to 0.5 km from the human habitations. During 2016, the district received 1471.75 mm rainfall from July to the end of October. Water accumulations in the paddy fields, which are good breeding grounds for the JE vector mosquitoes, were more during the outbreak period indicating a possible association between rice cultivation season and JE transmission. Mostly tribal villages of the district rear the pigs as it is a source of economy for them. In addition, high temperature and relative humidity prevailed in the district provided a suitable environment for JEV transmission. The district is also endemic for *falciparum* malaria since many decades (Gunasekaran et al. 2016). As the rice agro eco-system supports the breeding of the vectors of malaria (Sahu et al. 2017) and JE (Dwibedi et al. 2015), integrated vector management (IVM) is recommended to prevent the transmission of both the vector-borne diseases.

In summary, we confirmed the outbreak of JEV infection during 2016 in Malkangiri district, as JE virus was detected from *Cx. vishnui* collected from the outbreak affected villages. The environmental conditions, vector abundance, dusk index and HBI of JE vectors supported the occurrence of JE transmission in the district. Based on these findings, the appropriate vector control measures were recommended and the district administration implemented intense vector control activities as per the recommendations. The district has environmental risk for acquiring JE infection. This report of occurrence of JE in Malkangiri district points towards the need for public health vigilance and thorough screening of JE vectors regularly to prevent morbidity and mortality in future.
